# Characteristics of a Rollable Dielectric Barrier Discharge Plasma and Its Effects on Spinach-Seed Germination

**DOI:** 10.3390/ijms24054638

**Published:** 2023-02-27

**Authors:** Jun Sup Lim, Daeun Kim, Sehoon Ki, Sohail Mumtaz, Abdul Munnaf Shaik, Ihn Han, Young June Hong, Gyungsoon Park, Eun Ha Choi

**Affiliations:** 1 Plasma Bioscience Research Center (PBRC), Kwangwoon University, Seoul 01897, Republic of Korea; 2 Electrical and Biological Physics Department, Kwangwoon University, Seoul 01897, Republic of Korea; 3 Institute of Plasma Technology, Korea Institute of Fusion Energy, Gunsan 54004, Republic of Korea

**Keywords:** rollable dielectric barrier discharge (RDBD), omnidirectional and uniform treatment, plasma seed treatment, non-thermal atmospheric pressure plasma (NAP)

## Abstract

We investigated the characteristics of a rollable dielectric barrier discharge (RDBD) and evaluate its effects on seed germination rate and water uptake. The RDBD source was composed of a polyimide substrate and copper electrode, and it was mounted in a rolled-up structure for omnidirectional and uniform treatment of seeds with flowing synthetic air gas. The rotational and vibrational temperatures were measured to be 342 K and 2860 K, respectively, using optical emission spectroscopy. The chemical species analysis via Fourier-transform infrared spectroscopy and 0D chemical simulation showed that O_3_ production was dominant and NO_x_ production was restrained at the given temperatures. The water uptake and germination rate of spinach seeds by 5 min treatment of RDBD was increased by 10% and 15%, respectively, and the standard error of germination was reduced by 4% in comparison with the controls. RDBD enables an important step forward in non-thermal atmospheric-pressure plasma agriculture for omnidirectional seed treatment.

## 1. Introduction

Non-thermal atmospheric-pressure plasma (NAP) is an emerging technology with numerous applications in a variety of fields, including semiconductor manufacturing, medicine, agriculture, and environmental decontamination [1,2,3,4,5,6]. NAP contains a large number of reactive oxygen species (ROS) and reactive nitrogen species (RNS), which react with contamination particles, cells, bacteria, tumors, and metal surfaces [7,8,9]. Previously, NAP was widely used in the polymer and electronic industries for surface modification and the functionalization of various polymers. However, in recent years, the applications of NAP have expanded to include the treatment of biological matter, such as food, cancers, and viral diseases [10,11,12]. Plasma jets, surface dielectric barrier discharge (DBD), pin-to-plate-type plasma, and other plasma devices were subsequently developed based on the demand [13,14,15,16,17]. The development of surface DBD devices has progressed owing to their large surface area treatment for wound healing and seed treatments at low temperatures. The general design of DBDs restricts their use on straight surfaces because the curvature of the sample must also be considered. Furthermore, the size and shape of the seeds vary based on the plant species, which makes it difficult to provide uniform treatment using conventional devices. In this case, a flexible DBD device may offer an alternative; however, a device that can effectively overcome this difficulty has not yet been proposed. Recently, a flexible DBD was developed for sterilization purposes [18,19,20]. Kim et al., developed this inkjet-printed flexible DBD source, which inhibited the growth of fungi in blueberries [18]. Guo et al., studied the inactivation of a virus on film and the sterilization of natural bacteria using a flexible DBD source [19]. In particular, plasma treatment could remove the cuticle and epidermis layer on seeds, which improves water absorbance into the inner layers [21]. These flexible DBD devices may provide an alternative for overcoming the abovementioned limitations; however, to our knowledge, there are no existing reports on an omnidirectional design. Herein, we present the design of a novel rollable dielectric barrier discharge (RDBD) device that includes 200 micro-discharge cells for a uniform and omnidirectional plasma seed treatment. We investigated the rotational and vibrational temperatures using optical emission spectroscopy and performed Fourier-transform infrared (FTIR) spectroscopy and 0D (zero-dimension) chemical simulation analysis of plasma in the RDBD device. Furthermore, the proposed RDBD device was tested to assess the effect on the germination rate and water uptake of spinach seeds.

## 2. Results and Discussions

### 2.1. Physical and Chemical Properties of RDBD

**Figure 1:**
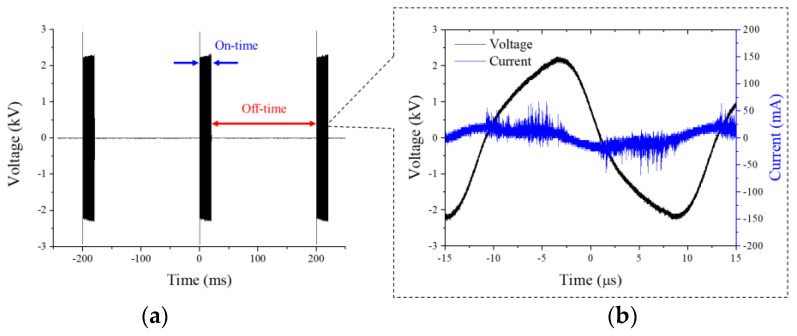
(**a**) Duty ratio of applied voltage, (**b**) voltage (black line) and discharge current (blue line) versus time.

The electrical characteristics of RDBD are shown in Figure 1. The duty ratio of the applied voltage with a sinusoidal wave on-time of 20 ms and an off-time of 180 ms at 0 V are shown in Figure 1a. A large number of discharge current peaks appear during the voltage period because the discharges of each of the 200 discharge cells in the RDBD device varied, as shown in Figure 1b. The root mean square voltage and discharge current were measured to be 1.8 kV and 2 mA, respectively. The electrical energy and dissipated power based on the duty ratio were measured to be 0.2 J and 2 mW, respectively.

**Figure 2:**
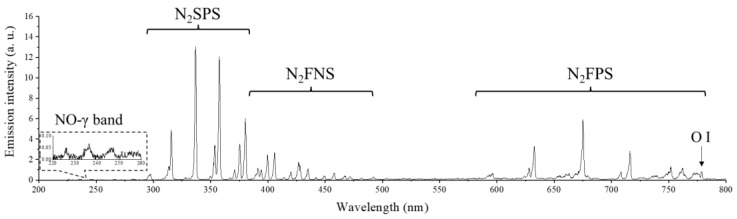
Optical emission spectrum of plasma on the RDBD surface.

The optical emission spectrum (OES) of the RDBD is depicted in Figure 2. The discharge emissions uniformly occurred at the discharge cells, and the OES results show that RDBD primarily emits the nitrogen band spectra, such as the N_2_ s positive system (N_2_SPS, 300–380 nm), N_2_ first negative system (N_2_FNS, 380–500 nm), and N_2_ first positive system (N_2_FPS, 500–800 nm). Additionally, the NO-γ band (220–260 nm) and atomic oxygen (778 nm) were observed in the OES. The dominant N_2_-band spectrum shows that RDBD was mainly discharged by nitrogen molecules.

**Figure 3:**
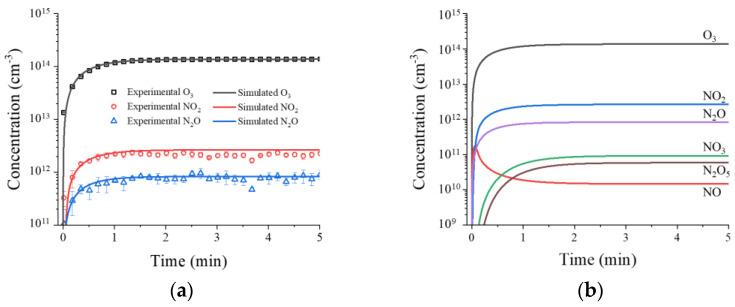
(**a**) Experimental and simulated results of O_3_, NO, and N_2_O versus time and (**b**) chemical species in 0D chemical simulation.

The rotational and vibrational temperatures were calculated via the spectrum-fitting method using the experimental spectrum of nitrogen molecules (337 nm), which was obtained from a fitted Boltzmann plot [22,23]. The rotational and vibrational temperatures were measured to be 342 K and 2860 K, respectively (see Supplementary Figure S1). The gas FTIR and 0D chemical simulation results are shown in Figure 3. In the FTIR spectroscopy results, O_3_, NO_2_, and N_2_O were increased for 1 min after the RDBD operation and were then kept stable. Their concentrations were $1.36\times 10^{14}{\;\mathrm{cm}}^{-3}$, $2.15\times 10^{12}{\;\mathrm{cm}}^{-3}$, and $8.25\times 10^{11}{\;\mathrm{cm}}^{-3}$, respectively, as shown in Figure 3a. Figure 3a,b show the chemical simulation results with the FTIR spectroscopy results and the chemical species in the 0D chemical simulation, respectively.

The crucial NO production was generated by reaction R6 in Table 1, which originated from vibrationally excited N_2_ [24]. However, owing to the low vibrational temperature of 2860 K, the NO concentration was simulated to a maximum of $1.66\times 10^{11}{\;\mathrm{cm}}^{-3}$ at 3 s and immediately decreased to $1.48\times 10^{10}{\;\mathrm{cm}}^{-3}$. The sharp decrease in NO occurred as the vibrational temperature approached its maximum at time, and the NO production in R6 was saturated. Then, reaction R7 occurred for NO_2_ formation from NO, which was effective at the low gas temperature. This led to a rapid decrease in NO concentration, as shown in Figure 3b. The NO, NO_3_, N_2_O_3_, N_2_O_4_, and N_2_O_5_ concentrations were too low to measure in the FTIR. The N_2_O_5_ concentration was calculated to be $8.0\times 10^{10}{\;\mathrm{cm}}^{-3}$ in this simulation. Moreover, N_2_O_3_ and N_2_O_4_ had very low calculated concentrations of $1.14\times 10^{4}{\;\mathrm{cm}}^{-3}$ and $1.0\times 10^{5}{\;\mathrm{cm}}^{-3}$, respectively. These results show that O_3_ production was dominant, owing to the low gas temperature in reaction R2.

### 2.2. Improvement of Water Uptake and Germination Rates in Seeds Using RDBD

**Figure 4:**
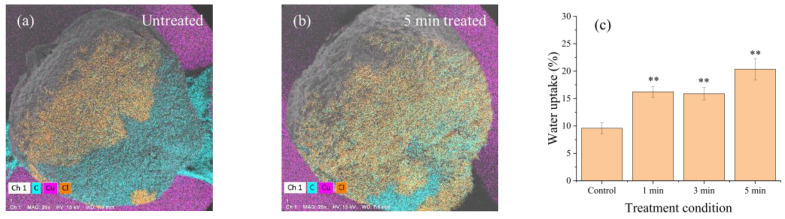
(**a**) EDS image of untreated spinach seed, (**b**) EDS image of spinach seed treated for 5 min, and (**c**) water uptake with RDBD treatment condition. The experiment was repeated three times with 150 seeds. (** denotes *p* < 0.01).

Figure 4 shows the energy dispersive X-ray spectroscopy (EDS) and water uptake results for spinach seeds that were untreated and treated by RDBD. To measure the range of water uptake on the seed surface, 1 μL of 10% NaCl solution was fed to the seed surface. Figure 4a,b show the EDS images of the seed surfaces, which depict the water absorption area by the 10% NaCl solution with an atomic Cl signal (orange color). The untreated seed exhibits the Cl signal over half of its area; however, the treated seed has a fully filled surface. These results show that the hydrophilicity of the spinach seed surface was enhanced by the RDBD treatment. The water uptake in the seeds is shown in Figure 4c based on seed weight. The seeds were treated for 1, 3, and 5 min by RDBD. The water uptake of the spinach seeds was enhanced by a maximum of 10% via the 5 min RDBD treatment.

**Figure 5:**
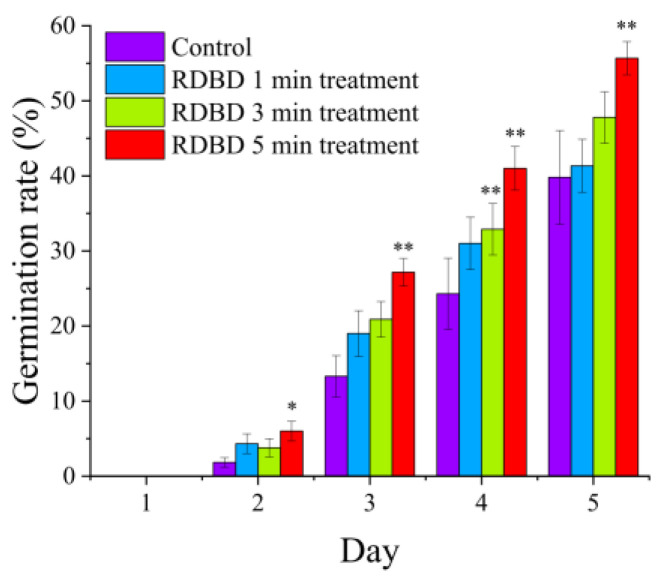
Spinach seed germination rate for each treatment day and treatment time. The experiment was repeated three times with 150 seeds. (* denotes *p* < 0.05 and ** denotes *p* < 0.01).

Figure 5 shows the seed germination rate of untreated and treated spinach seeds. A short treatment time of 1 min exhibited a similar germination rate to that of the control in this experiment. After 1 min of the RDBD operation, O_3_ production was too low to affect the seed, as shown in Figure 3a, and the result led to a lower rate of seed germination. Moreover, only RDBD treatments longer than 3 min exhibited a meaningful enhancement in seed germination, as shown in Figure 5. Notably, the 5 min RDBD treatment showed a significant increase in both water uptake and germination rate. Moreover, the standard errors of the seed germination were significantly reduced from 6.2% (control) to 2.2% (5 min RDBD treatment) in this experiment. Undoubtedly, O_3_ play a significant role in seed germination in this experiment. Here, the additional omnidirectional production of ROS enhanced the equal treatment of each seed inside the RDBD device. Furthermore, a relatively strong flow rate of 15 lpm in air gas can shake the seeds inside the RDBD device to increase the chance of the ROS mixing with the seeds. These characteristics led to an increase in seed germination and a decrease in the standard errors of germination in this experiment.

## 3. Materials and Methods

### 3.1. Rollable DBD Source

**Figure 6:**
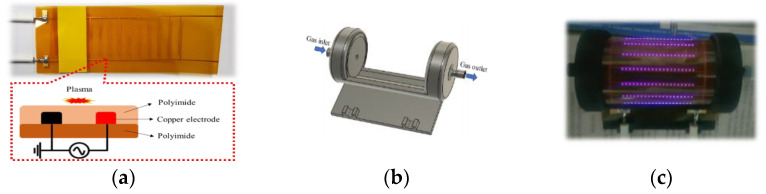
(**a**) Schematic of RDBD panel, (**b**) mount for RDBD, and (**c**) discharge in RDBD device.

The RDBD device was manufactured using a 200 µm-width polyimide substrate. Copper was inkjet-printed at the electrode on the polyimide film (total area: 100 × 200 mm), as shown in Figure 6a. To prevent the oxidation of the copper electrode, it was covered by polyimide again. The mount in Figure 6b for the RDBD device was fabricated using 3D printing with polylactic acid material.

The RDBD panel structure was rolled up with a 3D-printed mount. The plasma was operated using synthetic air (dry air, 21% O_2_ with N_2_ balance) gas of 15 lpm to remove the fluctuation of chemical species density by random moisture on natural air, and using an inverter that had a peak voltage of 2.2 kV with a 40 kHz sinusoidal wave. Discharge was intended to occur on the inner surface of the RDBD device in the mount. To avoid burning the polyimide surface owing to thermal damage from plasma, the applied voltage was applied using a duty ratio of 10%, as shown in the following expression:

(1) $$\mathrm{Duty}\;\mathrm{ratio}\left(\%\right)=\frac{\mathrm{on}\;\mathrm{time}}{\mathrm{on}\;\mathrm{time}+\mathrm{off}\;\mathrm{time}}\times 100$$

where on-time refers to the sinusoidal voltage signal, and off-time is the rest time of the voltage corresponding to 0 V for cooling. The on-time was fixed at 20 ms, and the off-time was applied for 180 ms in this experiment.

### 3.2. Electrical and Optical Properties of Rollable DBD

The electrical characteristic of the device was measured using a high-voltage probe (P6015A, Tektronix, Beaverton, OR, USA) and current probe (P6021, Tektronix, Beaverton, OR, USA) that was connected to an oscilloscope (DSOX3104T, Keysight, Santa Rosa, CA, USA) for data recording as a function of time. The electrical energy I and dissipated power (*P*) were calculated using the following expressions:

(2) $$E=\int_{0}^{T}v\left(t\right)i\left(t\right)\mathrm{dt}\;[J],\;$$

(3) $$P=\mathrm{Duty}\;\mathrm{ratio}\;\times \frac{1}{T}\int_{0}^{T}v\left(t\right)i\left(t\right)\mathrm{dt}\;[W],\;$$

where v(t) and i(t) are the voltage and current versus time from the recorded data, respectively, and T is the period of voltage. The optical-emission property of RDBD was measured using a charge-coupled device (PIMAX4, Princeton instruments Inc., Trenton, NJ, USA) with a monochromator (SP2750i, Princeton instruments Inc., Trenton, NJ, USA). The gate width of the charge-coupled device was set to 1 s and five times of accumulation. The optical fiber was set to 5 mm away from the outer RDBD panel. To measure the optical emission profile of RDBD, the wavelength range of 200–800 nm was recorded with 150-groove grating. To measure the spectrum of rotational structure in N_2_SPS, the wavelength range of 334–337.5 nm was recorded with 1200-groove grating.

### 3.3. Chemical Species Measurement in FTIR Spectroscopy

The chemical species production of the RDBD device was measured using FTIR spectroscopy (Martix-MG5, Bruker, Billerica, MA, USA). A 1 m Teflon tube was connected between the gas inlet of the FTIR spectroscope and the gas outlet of the RDBD device. The time interval of the FTIR spectroscopy was set to 10 s, and the wave number ranged from 600 to 3000 cm^−1^ in this experiment. Concentrations of chemical species (n_species_) were calculated using the Beer–Lambert law with the following absorption cross-section (σ) [37]:

(4) $$n_{\mathrm{species}}=-\frac{1}{\sigma l}\ln\left(\frac{I_{\mathrm{Trans}}}{I_{\mathrm{BG}}}\right),$$

(5) $$\sigma_{O_{3}}=6.51\times 10^{-19}{\;\mathrm{cm}}^{2}\;\mathrm{at}\;1054.65{\;\mathrm{cm}}^{-1},$$

(6) $$\sigma_{{\mathrm{NO}}_{2}}=2.32\times 10^{-18}{\;\mathrm{cm}}^{2}\;\mathrm{at}\;1631.81{\;\mathrm{cm}}^{-1},$$

(7) $$\sigma_{N_{2}O}=3.34\times 10^{-18}{\;\mathrm{cm}}^{2}\;\mathrm{at}\;2239.07{\;\mathrm{cm}}^{-1},$$

where σ is the absorption cross-section area, and l is the optical path length (500 cm) in the FTIR spectroscope. I_Trans_ is the measured transmittance in the FTIR spectroscope, which corresponded to the wave number of the absorption peak. I_BG_ is the background signal of the FTIR spectroscope.

### 3.4. Zero-Dimension (0D) Chemical Species Simulation

A 0D simulation of the chemical species was used to understand the chemical reactions in the RDBD device. In this simulation, 51 reactions were used with 10 species, as shown in Table 1.

The time-dependent continuity equation is used in this simulation [24,38]:

(8) $$\frac{\partial n_{i}}{\partial t}=\sum_{j}k_{j}^{S}\prod n_{r,j}-\sum_{j}k_{j}^{L}\prod n_{r,j}-\frac{n_{i}}{\tau_{\mathrm{dif}}}$$

where $k_{j}^{S}$ and $k_{j}^{L}$ are the rate coefficients of the source and loss reactions, respectively. $n_{r,j}$ are the concentrations of each chemical species involved in the reaction [38]. $\tau_{\mathrm{dif}}$ is the time constant of the diffusion loss owing to the air flow in the RDBD device [24]. The diffusion owing to the air flow partially contributes to the concentration of the chemical species that is produced, and the time constant of the diffusion loss ($\tau_{\mathrm{dif}}$) in this simulation was considered to be 30 s. The concentration of vibrationally excited N_2_ ($n_{n_{2}\left(v\right)}$) is determined by the following expressions [24]:

(9) $$n_{N_{2}\left(v\right)}=n_{N_{2}}F_{v>12}=n_{N_{2}}\exp\left(-\frac{12\Delta \varepsilon_{v}}{k_{b}T_{v}}\right),$$

(10) $$T_{v}=T_{g}+T_{v}^{0}\left[1-\exp\left(-t/\tau_{v}\right)\right],$$

where $k_{b}$ is the Boltzmann constant. $\Delta \varepsilon_{v}$ (=0.29 eV) is the vibrationally excited nitrogen at the level above v = 12 that contributes to NO production in R6. $T_{v}$ is the vibrational temperature, and $T_{v}^{0}$ (=2860 K) is the steady state of the vibrational temperature. $\tau_{v}$ (=0.01 s) is the time constant of the vibrational temperature increase, and $T_{g}$ (=342 K) is the gas temperature. In this simulation, the measured rotational temperature from the N_2_ spectrum was used as a gas temperature under the assumption of the rotational–translational temperature equilibrium [39,40]. The initial concentrations of all species, except for atomic oxygen, excited N_2_ and O_2_, which were set to $1.0\times 10^{4}{\;\mathrm{cm}}^{-3}$. The excited species parameters,$n_{O}=7.6\times 10^{7}{\;\mathrm{cm}}^{-3}$, $n_{O\left(D\right)}=8.0\times 10^{7}{\;\mathrm{cm}}^{-3}\;$, $n_{O_{2}\left(a\right)}=1.0\times 10^{7}{\;\mathrm{cm}}^{-3}$, and $n_{N_{2}\left(A\right)}=4.0\times 10^{7}{\;\mathrm{cm}}^{-3}$, were set to constants over the simulation period. The equations with the reactions were solved using the ordinary-differential-equation solver in MATLAB (MathWorks, Natick, MA, USA).

### 3.5. Spinach Seed Treatment and Germination

Spinach seeds were soaked in deionized (DI) water for 24 h before treatment. Additionally, the seeds were inserted into the mount and covered by the surrounding rolled-up RDBD panel, as shown in Figure 6c (Also see Supplementary Materials Figure S3). Each treatment condition was performed with 150 seeds for 1, 3, or 5 min. Treated seeds were divided into groups of 50 seeds per 90 mm petri dish, which had three pieces of filter paper. The filter paper was supplied with 3 mL DI water on the first day of the experiment, and 1 mL DI water was additionally supplied on the third day. The seeds were covered in the petri dish and grown in an incubator at 26 °C with 60% humidity. Seed germination was considered successful if the seed grew a stem of more than 1 mm. The germination rate was calculated using the following expression:

(11) $$\mathrm{Germination}\;\mathrm{rate}\;\left(\%\right)=\frac{N_{G}}{N_{T}}\times 100\%$$

where $N_{T}$ is the total number of seeds in a petri dish, and $N_{G}$ is the total number of germinated seeds in a petri dish.

### 3.6. Measurement of Water Uptake of Seeds

The water uptake was measured two ways. To measure the water absorption area on the seed surface, 1 μL of 10% NaCl solution was fed to the seed surface during 10 min. Energy dispersive X-ray spectroscopy (EDS) was used to measure the absorption area by the Cl signal of absorbed NaCl solution in the seed surface.

The water absorption rate ($W_{a}$) was measured by DI water absorption in the seed. After plasma treatment, seeds were incubated (26 °C with 60% humidity) in the petri dish with wet filter paper for 24 h. The filter paper was fed 3 mL of DI water. After 24 h, the weight W_a_ was measured using the following expression [41]:

(12) $$W_{a}\left(\%\right)=\frac{W_{1}-W_{o}}{W_{o}}\times 100\%,\;$$

where W_o_ is the weight of 50 seeds before the plasma treatment, and W_1_ is the corresponding weight 24 h after the plasma treatment.

### 3.7. Statistical Analysis

The analysis of the variance of the differences among samples used standard errors in this report. Experimental results were repeated three times. The seed germination was statistically analyzed by Student’s *t*-test, and significant differences were indicated based on *p* < 0.05. (* denotes *p* < 0.05 and ** denotes *p* < 0.01).

## 4. Conclusions

NAP is a new technology with a wide range of applications in different fields, including agriculture. Innovative NAP devices that are designed with improved understanding and usability are desperately needed to advance NAP applications in agriculture. In this study, we developed and investigated the properties of an RDBD device that is operated using synthetic air gas. The rotational and vibrational temperatures were measured as 342 K and 2860 K, respectively. The rotational temperature results show that the RDBD device is safe and efficient for use in agricultural fields. The N_2_, O, and NO-γ spectra were observed using optical emission spectroscopy. The O_3_, NO_2_, and N_2_O productions of the RDBD device were measured to be $1.36\times 10^{14}{\;\mathrm{cm}}^{-3}$, $2.15\times 10^{12}{\;\mathrm{cm}}^{-3}$, and $8.25\times 10^{11}{\;\mathrm{cm}}^{-3}$, respectively, based on FTIR absorption spectroscopy. The 0D chemical simulation and experimental FTIR spectroscopy results were shown to be dominant for O_3_, whereas the nitrogen species were recessive, owing to their low temperatures in RDBD. The proposed RDBD device was also used to assess seed germination and seed water uptake. When 150 spinach seeds were simultaneously treated with RDBD, the water uptake and germination increased by 10% and 15%, respectively, after a 5 min RDBD treatment. The RDBD device exhibited an enhancement in the germination rate, as well as a reduction in the standard errors of the germination rate owing to omnidirectional seed treatment. These findings represent a significant advancement in NAP devices, which may help in advancing the potential applications of plasma in agriculture.

## Figures and Tables

**Table 1:** Chemical reaction list used in simulation.

No.	Reaction	Rate Coefficient (cm^3^/s or cm^6^/s)	Ref
R1	$O+O+M\to O_{2}+M$	$4.5\times 10^{-34}\exp\left(630/T_{g}\right)$	[25]
R2	$O+O_{2}+M\to O_{3}+M$	$5.6\times 10^{-34}{\left(T_{g}/300\right)}^{-2.8}$	[25]
R3	$O+O_{3}\to O_{2}+O_{2}$	$8.0\times 10^{-12}\exp{\left(-2060/T_{g}\right)}^{-1.6}$	[25]
R4	$O+N_{2}+M\to N_{2}O+M$	$3.9\times 10^{-35}\exp\left(-10,400/T_{g}\right)\;$	[26]
R5	$O+N_{2}\left(A^{3}\Sigma \right)\to \mathrm{NO}+N{(}^{2}D)\;$	$7.0\times 10^{-12}$	[27]
R6	$O+N_{2}\left(\nu \right)\to \mathrm{NO}+N$	$1.0\times 10^{-11}$	[27]
R7	$O+\mathrm{NO}+M\to {\mathrm{NO}}_{2}+M$	$1.0\times 10^{-31}{\left(T_{g}/300\right)}^{-1.6}$	[25]
R8	$O+\mathrm{NO}\to {\mathrm{NO}}_{2}$	$4.2\times 10^{-18}$	[26]
R9	$O+{\mathrm{NO}}_{2}\to \mathrm{NO}+O_{2}$	$5.5\times 10^{-12}\exp\left(188/T_{g}\right)$	[28]
R10	$O+{\mathrm{NO}}_{2}+M\to {\mathrm{NO}}_{3}+M$	$1.31\times 10^{-31}{\left(298/T_{g}\right)}^{1.5}$	[28]
R11	$O+{\mathrm{NO}}_{2}\to {\mathrm{NO}}_{3}$	$2.3\times 10^{-11}{\left(T_{g}/300\right)}^{0.24}$	[28]
R12	$O+{\mathrm{NO}}_{3}\to O_{2}+{\mathrm{NO}}_{2}$	$1.7\times 10^{-11}$	[25]
R13	$O+N_{2}O\to \mathrm{NO}+\mathrm{NO}$	$1.5\times 10^{-16}\exp\left(-14,090/T_{g}\right)$	[26]
R14	$O+N_{2}O_{5}\to \mathrm{products}$	$3\times 10^{-16}$	[26]
R15	$O_{2}+O_{2}\to O+O_{3}$	$2.0\times 10^{-11}\exp\left(-49,800/T_{g}\right)$	[26]
R16	$O{(}^{1}D)+N_{2}+N_{2}\to N_{2}O+N_{2}$	$9.0\times 10^{-37}$	[29]
R17	$O_{3}+\mathrm{NO}\to {\mathrm{NO}}_{2}+O_{2}$	$1.8\times 10^{-12}\exp\left(-1370/T_{g}\right)$	[25]
R18	$O_{3}+{\mathrm{NO}}_{2}\to {\mathrm{NO}}_{3}+O_{2}$	$1.2\times 10^{-13}\exp\left(-2450/T_{g}\right)$	[30]
R19	$O_{3}+M\to O+O_{2}+M$	$7.26\times 10^{-10}\exp\left(-11,400/T_{g}\right)$	[31]
R20	$O_{3}+O_{2}\left(a^{1}\Delta \right)\to O+O_{2}+O_{2}$	$5.2\times 10^{-11}\exp\left(-2840/T_{g}\right)$	[25]
R21	N+O+M→NO+M	$6.3\times 10^{-33}\exp\left(140/T_{g}\right)$	[25]
R22	$N+O_{2}\to \mathrm{NO}+O$	$1.5\times 10^{-14}T_{g}\exp\left(-3270/T_{g}\right)$	[32]
R23	$N+O_{3}\to \mathrm{NO}+O_{2}$	$5.0\times 10^{-22}$	[33]
R24	$N+N+M\to N_{2}+M$	$8.3\times 10^{-34}\exp\left(500/T_{g}\right)$	[26]
R25	$N+\mathrm{NO}\to N_{2}+O$	$2.1\times 10^{-11}\times \exp\left(100/T_{g}\right)$	[25]
R26	$N+{\mathrm{NO}}_{2}\to N_{2}O+O$	$5.8\times 10^{-12}\exp\left(220/T_{g}\right)$	[25]
R27	$N+{\mathrm{NO}}_{2}\to N_{2}+O+O$	$9.1\times 10^{-13}$	[34]
R28	$N+{\mathrm{NO}}_{2}\to \mathrm{NO}+\mathrm{NO}$	$6.0\times 10^{-13}$	[34]
R29	$N+{\mathrm{NO}}_{2}\to N_{2}+O_{2}$	$7.0\times 10^{-13}$	[34]
R30	$N_{2}+O_{2}\to O+N_{2}O$	$2.5\times 10^{-10}\exp\left(-50,390/T_{g}\right)$	[26]
R31	$N_{2}+M\to N+N+M$	$5.4\times 10^{-8}\left[1-\exp\left(-3354/T_{g}\right)\right]\exp\left(-113,200/T_{g}\right)$	[26]
R32	$\mathrm{NO}+{\mathrm{NO}}_{2}+M\to N_{2}O_{3}+M$	$3.1\times 10^{-34}{\left(T_{g}/300\right)}^{-7.7}$	[28]
R33	$\mathrm{NO}+{\mathrm{NO}}_{3}\to {\mathrm{NO}}_{2}+{\mathrm{NO}}_{2}$	$1.59\times 10^{-11}\exp\left(122/T_{g}\right)$	[33]
R34	$\mathrm{NO}+\mathrm{NO}\to N+{\mathrm{NO}}_{2}$	$3.3\times 10^{-16}\left(300/T_{g}\right)\exp\left(-39,200/T_{g}\right)$	[26]
R35	$\mathrm{NO}+O_{2}\to O+{\mathrm{NO}}_{2}$	$2.8\times 10^{-12}\exp\left(-23,400/T_{g}\right)$	[26]
R36	${\mathrm{NO}}_{2}+{\mathrm{NO}}_{2}+M\to N_{2}O_{4}+M$	$1.44\times 10^{-32}\exp{\left(110/T_{g}\right)}^{3.8}$	[28]
R37	${\mathrm{NO}}_{2}+{\mathrm{NO}}_{3}+M\to N_{2}O_{5}+M$	$3.7\times 10^{-30}{\left(300/T_{g}\right)}^{4.1}$	[28]
R38	${\mathrm{NO}}_{2}+{\mathrm{NO}}_{3}\to {\mathrm{NO}}_{2}+\mathrm{NO}+O$	$2.3\times 10^{-13}\exp\left(-1600/T_{g}\right)$	[26]
R39	${\mathrm{NO}}_{2}+{\mathrm{NO}}_{2}\to \mathrm{NO}+\mathrm{NO}+O_{2}$	$3.3\times 10^{-12}\exp\left(-13,500/T_{g}\right)$	[26]
R40	${\mathrm{NO}}_{2}+O_{2}\to \mathrm{NO}+O_{3}$	$2.8\times 10^{-12}\exp\left(-25,400/T_{g}\right)$	[26]
R41	${\mathrm{NO}}_{2}+M\to \mathrm{NO}+O+M$	$6.8\times 10^{-6}{\left(300/T_{g}\right)}^{2}\exp\left(-36,180/T_{g}\right)$	[26]
R42	${\mathrm{NO}}_{2}+N_{2}\left(A^{3}\Sigma \right)\to N_{2}+\mathrm{NO}+O$	$1.3\times 10^{-11}$	[25]
R43	${\mathrm{NO}}_{3}+O_{2}\to {\mathrm{NO}}_{2}+O_{3}$	$1.5\times 10^{-12}\exp\left(-15,020/T_{g}\right)$	[26]
R44	${\mathrm{NO}}_{3}+M\to {\mathrm{NO}}_{2}+O+M$	$3.1\times 10^{-5}{\left(300/T_{g}\right)}^{2}\exp\left(-25,000/T_{g}\right)$	[26]
R45	${\mathrm{NO}}_{3}+M\to \mathrm{NO}+O_{2}+M$	$6.2\times 10^{-5}{\left(300/T_{g}\right)}^{2}\exp\left(-25,000/T_{g}\right)$	[26]
R46	${\mathrm{NO}}_{3}+{\mathrm{NO}}_{3}\to {\mathrm{NO}}_{2}+{\mathrm{NO}}_{2}+O_{2}$	$4.3\times 10^{-12}\exp\left(-3850/T_{g}\right)$	[35]
R47	$N_{2}O_{3}+M\to \mathrm{NO}+{\mathrm{NO}}_{2}+M$	$1.9\times 10^{-7}{\left(T_{g}/300\right)}^{-8.7}\exp\left(-4880/T_{g}\right)$	[28]
R48	$N_{2}O_{4}+M\to {\mathrm{NO}}_{2}+{\mathrm{NO}}_{2}+M$	$1.3\times 10^{-5}{\left(T_{g}/300\right)}^{-3.8}\exp\left(-6400/T_{g}\right)$	[28]
R49	$N_{2}O_{5}+M\to {\mathrm{NO}}_{2}+{\mathrm{NO}}_{3}+M$	$1.3\times 10^{-3}{\left(T_{g}/300\right)}^{-3.5}\exp\left(-11,000/T_{g}\right)$	[28]
R50	$N_{2}O+N_{2}\left(A^{3}\Sigma \right)\to O+N_{2}+N_{2}$	$8.0\times 10^{-11}$	[36]
R51	$N_{2}O+N_{2}\left(A^{3}\Sigma \right)\to \mathrm{NO}+N+N_{2}$	$8.0\times 10^{-11}$	[36]

M is N_2_ or O_2_. T_g_ is the gas temperature (K).

## Data Availability

Please contact the corresponding author to discuss the availability of the data presented in this study.

## Supplementary Materials

The following supporting information can be downloaded at: https://www.mdpi.com/article/10.3390/ijms24054638/s1.
