# Supplementary material for: Characteristics of a Rollable Dielectric Barrier Discharge Plasma and Its Effects on Spinach-Seed Germination
Source: Int J Mol Sci. 2023 Feb 27;24(5):4638. doi: 10.3390/ijms24054638 (PMC10002516; doi:10.3390/ijms24054638)
Supplement: Supplementary file 1 [file ijms-24-04638-s001.zip › ijms-2198304-supplementary.pdf]

Supporting Information

Characteristics of a rollable DBD plasma device and its effects on spinach seed germination

Jun Sup Lim[1], Daeun Kim[2], Sehoon Ki[3], Sohail Mumtaz[2], Abdul Munnaf Shaik[2], Ihn Han[1], Young June Hong[1], Gyungsoon Park[2], Eun Ha Choi[1,2]

[1]*Plasma Bioscience Research Center (PBRC), Kwangwoon University, Seoul 01897, Republic of Korea*
[2]*Electrical and biological physics department, Kwangwoon University, Seoul 01897, Republic of Korea*
[3]*Institute of plasma technology, Korea institute of fusion energy, Gunsan 54004, Republic of Korea*

* Corresponding author, E-mail address: ehchoi@kw.ac.kr

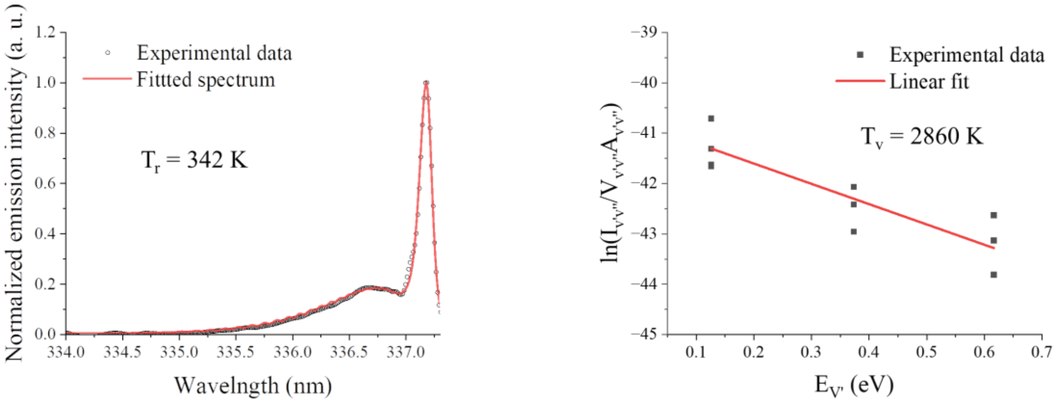

(a) (b)

Figure S1. (a) Experimental and simulated spectrum for rotational temperature in nitrogen second positive system in 337 nm ($C^3\Pi_u(\nu = 0) \rightarrow B^3\Pi_g(\nu = 0)$), (b) Boltzmann plot for vibrational temperature

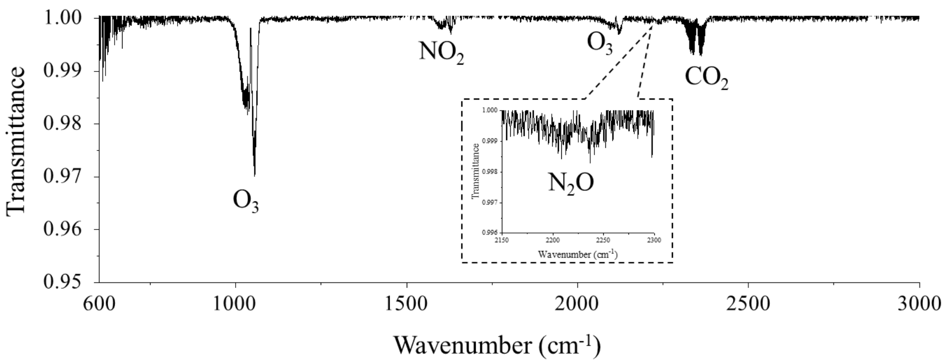

Figure S2. Transmittance result of RDBD by FTIR

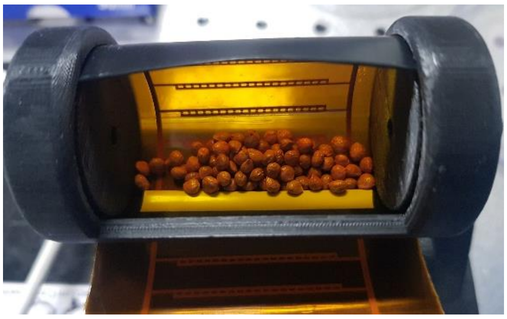

Figure S3. RDBD with mount for seed treatment
